# Time to Treatment Intensification to Reduce Diabetes-Related Complications: A Post Hoc Study

**DOI:** 10.3390/healthcare10091673

**Published:** 2022-09-01

**Authors:** Piranee Kaewbut, Natapong Kosachunhanun, Arintaya Phrommintikul, Dujrudee Chinwong, John J. Hall, Surarong Chinwong

**Affiliations:** 1 PhD’s Degree Program in Pharmacy, Faculty of Pharmacy, Chiang Mai University, Chiang Mai 50200, Thailand; 2 Department of Pharmaceutical Care, School of Pharmaceutical Sciences, University of Phayao, Phayao 56000, Thailand; 3 Department of Internal Medicine, Faculty of Medicine, Chiang Mai University, Chiang Mai 50200, Thailand; 4 Department of Pharmaceutical Care, Faculty of Pharmacy, Chiang Mai University, Chiang Mai 50200, Thailand; 5 Center of Excellence for Innovation in Analytical Science and Technology for Biodiversity-Based Economic and Society (I-ANALY-S-T_B.BES-CMU), Chiang Mai University, Chiang Mai 50200, Thailand; 6 School of Public Health and Community Medicine, University of New South Wales, Sydney, NSW 2052, Australia

**Keywords:** clinical inertia, therapeutic inertia, time to treatment intensification, diabetes-related complications, diabetic nephropathy

## Abstract

Patients with type 2 diabetes mellitus (T2DM) can be affected by clinical inertia, leading to abysmal results. Studies on a suitable timeframe for treatment intensification remain scarce—especially outside of developed countries. This study aimed to explore the association between time to treatment intensification and diabetes-related complications. A database from a tertiary care hospital in Thailand was retrieved in order to conduct a retrospective cohort study for the years 2011–2017. This study comprised outpatients with T2DM presenting an HbA1c of ≥7.0%. Eligible patients were divided into three groups based on the time of treatment intensification: no delayed treatment intensification, treatment intensification within 6 months, and treatment intensification after 6 months. A Cox proportional hazards model was used to investigate the association between time to treatment intensification and diabetes-related complications. A total of 686 patients were included in the final analysis. During 6.5 years of median follow-up, the group with treatment intensification within 6 months was more strongly associated with diabetic nephropathy compared to the group with no delayed treatment intensification (adjusted HR 2.35; 95%CI 1.35–4.09). Our findings reveal that delaying treatment intensification by even 6 months can increase the likelihood of diabetic nephropathy compared to no delayed treatment intensification. We suggest that patients with T2DM whose blood glucose levels are outside the target range promptly receive treatment intensification.

## 1. Introduction

Clinical inertia is a synonym for therapeutic inertia [1,2] and is defined as the failure to initiate or escalate medication when needed [3,4,5,6]. Clinical inertia is a significant factor contributing to the inadequate therapy of chronic diseases (e.g., diabetes, hypertension, and dyslipidemia) [7,8]. In the U.S., a study revealed that approximately 70.0% of patients with T2DM had experienced clinical inertia [9]. Similarly, a study in Thailand found that 26.2 to 68.4% of patients with T2DM had experienced clinical inertia [10,11,12]. This phenomenon is becoming a greater concern in the global management of diabetes [13,14]. Clinical inertia arises at all stages of diabetes treatment [8] and is a major factor in obtaining and maintaining optimal glycemic control [15,16]. Moreover, clinical inertia can lead to cardiovascular complications, progressing to diabetic retinopathy and diabetic nephropathy [11,17,18,19].

The American Diabetes Association (ADA) 2022 and the Thai Clinical Practice Guideline 2017 recommend that adults with diabetes and without complications or comorbidities aim for a strict HbA1c target lower than 6.5%. However, this strict target increases the risk of hypoglycemia, which can potentially be fatal. As a result, most people with diabetes aim for an HbA1c of <7.0% [20,21]. Lifestyle modifications, exercise, and diet control should be advised first for patients with diabetes. Pharmacologic treatment is considered next depending on comorbidities, patient-centered treatment variables, and management requirements when the HbA1c level cannot be controlled within the target range [20]. If the glycemic target has not been reached after three months of treatment initiation, treatment intensification with other antidiabetic drugs is considered [20,21].

Surprisingly, a U.K. study found that the median time from being above the HbA1c cutoff to intensification with an additional oral antidiabetic drug (OAD) was 2.9, 1.9, or 1.6 years among individuals with HbA1c of ≥7.0, ≥7.5, or ≥8.0%, respectively [22]. A study in Japan found that patients receiving three OADs had a median time from an HbA1c of ≥7.0% to intensification with OAD, glucagon-like peptide-1 receptor agonist, or insulin of 8.1, 9.1, or 6.7 months, respectively [23]. In Thailand, a retrospective cohort study examining the electronic medical records of newly diagnosed type 2 diabetes patients in 16 community hospitals in Ubon Ratchathani found that patients obtaining medical services from 1 June 2008 to 31 December 2010 (phase 1), from 1 January 2011 to 31 December 2013 (phase 2), and from 1 January 2014 to 31 December 2015 (phase 3) had average times from treatment initiation to first clinical inertia of 377.7 ± 5.5, 348.8 ± 3.4, and 316.8 ± 3.6 days, respectively [12]. 

Our previously published study demonstrated that clinical inertia (also as a result of delayed treatment intensification) had a significant effect on diabetic nephropathy [17]. However, information on the effect of time to treatment intensification on diabetes-related complications remains limited. Therefore, in this study, we conducted a post hoc analysis of our published study to explore the effect of time to treatment intensification on diabetes-related complications.

## 2. Materials and Methods

### 2.1. Study Design and Setting

This study constitutes a retrospective record-based study conducted at Maharaj Nakorn Chiang Mai Hospital, a tertiary teaching institute in northern Thailand, from 1 January 2011 to 31 December 2011 with follow-up to 31 December 2017. The study was conducted according to the Declaration of Helsinki. Research Ethics Committee 4 of the Faculty of Medicine, Chiang Mai University approved this study on 25 January 2018. The serial number of the Ethics Board decision was 030/2018.

### 2.2. Study Participants

We reviewed 6033 charts of outpatients with T2DM receiving antihyperglycemic therapy. The study included participants with T2DM aged 40 to 65 years and presenting an HbA1c of ≥7.0%. Participants meeting any of the following criteria were excluded: (1) having a history of symptomatic hypoglycemia, (2) pregnant or breastfeeding, (3) in end-of-life care, (4) relying solely on insulin, (5) having many comorbidities (Charlson comorbidity index (CCI) score of ≥3), (6) with documented poor lifestyle modifications and medication adherence, (7) with no blood sugar results, or (8) under referral to other hospitals. 

**Figure 1:**
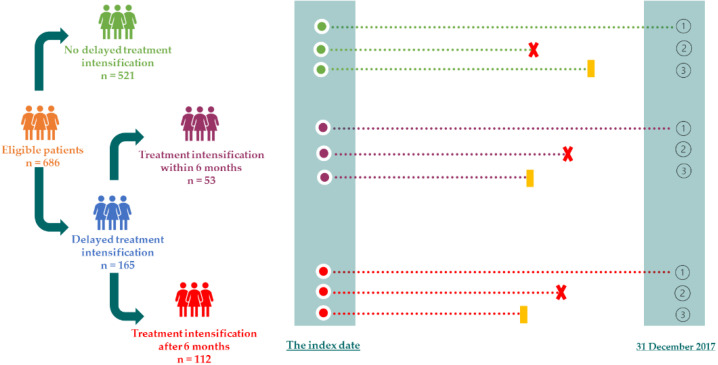
Study protocol. Patients were classified into three groups based on the time of treatment intensification, then the patients were followed up for diabetes-related complications from the index date to 31 December 2017. ➀ The patient had no diabetes-related complications until the end date of the follow-up; ➁ The patient was lost to follow-up; ➂ The patient had diabetes-related complications. 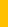 The first event of diabetes-related complications. 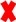 Loss to follow-up.

Eligible patients were divided into three groups by time to treatment intensification (TTTI): no delayed treatment intensification, treatment intensification within six months, and treatment intensification after six months (Figure 1). 

TTTI was calculated by subtracting the index date from the first date of treatment intensification. 

The index date was defined as the date of the first HbA1c laboratory test above the target level (HbA1c < 7.0%).

Treatment intensification was characterized as increasing the dosage of existing antidiabetic drugs, changing from an OAD to insulin or glucagon-like peptide-1 receptor (GLP-1) agonists, or escalating new antidiabetic drugs without discontinuing or reducing the dose of other antidiabetic drugs. 

No delayed treatment intensification was defined as patients with HbA1c ≥ 7.0% receiving treatment intensification at the index date or the consequent prescription.

Delayed treatment intensification was defined as patients with HbA1c ≥ 7.0% not receiving treatment intensification at the index date and the consequent prescription. Patients experiencing delayed treatment intensification were divided into two groups: patients receiving treatment intensification within six months and patients receiving treatment intensification after six months.

### 2.3. Data Collection

Eligible patients had their demographic, clinical, and laboratory characteristics reviewed, focusing on individual glycemic goals, treatment intensification, renal function, primary prevention of cardiovascular disease therapy, and time to treatment intensification. This included information on laboratory tests for hemoglobin A1c (HbA1c) and low-density lipoprotein cholesterol (LDL-C), cardioprotective medications, and diabetes-related complications. This information was recorded for eligible patients from the index date until the date the patient’s medical record was finished or 31 December 2017.

### 2.4. Study Outcomes

Patients were followed up from the index date until the first occurrence of diabetes-related complications, or until the end date of the follow-up, whichever came first, or until the patient’s medical record was finished. The time for diabetes-related complications was defined as the time between the index date and the first diabetes-related complications.

The primary outcome was diabetes-related complications, which are a composite of macrovascular and microvascular complications.

The secondary outcomes included (1) a composite of macrovascular complications: the first occurrence of myocardial infarction (MI), stroke, or heart failure (HF) or (2) a composite of microvascular complications: the first occurrence of diabetic nephropathy (DN) or diabetic retinopathy (DR).

### 2.5. Statistical Analysis

Stata Software, version 14 was used to analyze and compute all statistical data. A *p*-value of <0.05 was considered statistically significant in the two-tailed tests. The categorical variables are described as counts and percentages. Continuous variables are described as the mean ± standard deviation (SD) or median (interquartile range, IQR). Fisher’s exact test was used to compare differences between groups for categorical variables. Analysis of variance (ANOVA) or Kruskal–Wallis test was used to determine the differences between groups for continuous variables, as appropriate. Univariable Cox’s regressions were used to assess the effect of time to treatment intensification on diabetes-related complications. Multivariable Cox’s regression was used to control confounding factors. Confounding factors included age, sex, smoking status, duration of T2DM, CCI score, HbA1c, LDL-C, blood pressure, and cardio-protective medications (aspirin, ACEI/ARB). These factors were chosen based on previously published research [17] and our clinical experience with T2DM patients. Schoenfeld’s global test method was used to test the proportional hazards assumption. 

## 3. Results

### 3.1. Baseline Characteristics

**Figure 2:**
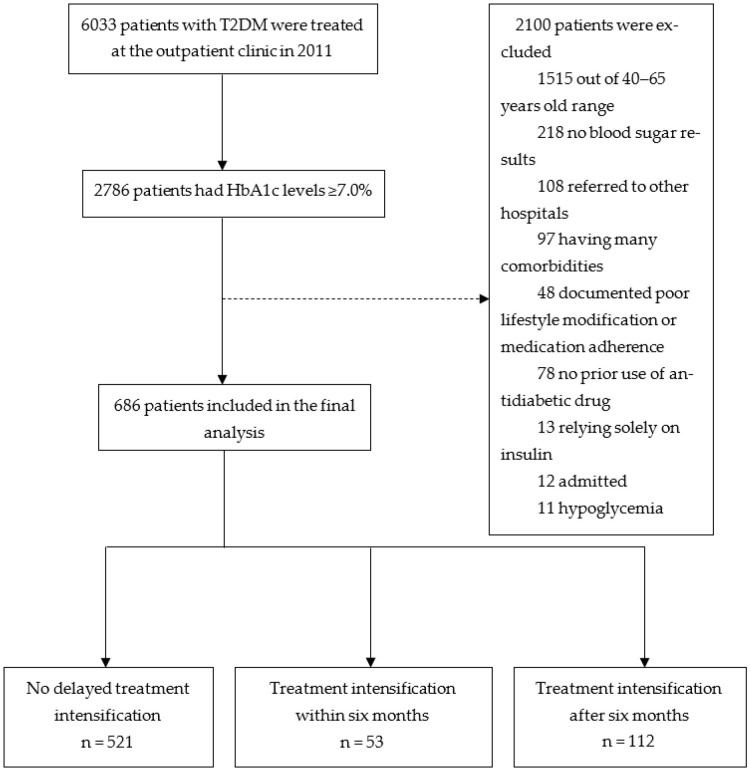
Patient flow diagram.

From 1 January 2011 to 31 December 2011, the medical records of 6033 patients with T2DM were reviewed. The final analysis comprised 686 patients (Figure 2). Of these, 521 patients were placed in the no delayed treatment intensification group, whereas 165 patients were placed in the delayed treatment intensification groups (53 patients receiving treatment intensification within six months and 112 patients receiving treatment intensification after six months).

In this cohort, the mean age of patients was 53.6 ± 6.0 years; 389 (56.7%) patients were male; and the median (IQR) baseline HbA1c was 7.9% (7.3 to 9.0). The median (IQR) duration since T2DM diagnosis was 5.0 (3.0 to 6.0) years. About 71.0% presented with hypertension, and 4.5% were current smokers. Patients with a history of cardiovascular disease, DN, and DR accounted for 7.7, 10.1, and 7.7% of patients, respectively (Table 1). A comparison between the three groups in the final analysis showed a significant difference in the history of DR and HbA1c at baseline. The no delayed treatment intensification group had a history of DR and HbA1c at baseline higher than that in the delayed treatment intensification group (all *p*-values < 0.05).

### 3.2. Association between Time to Treatment Intensification and Diabetes-Related Complications 

In our study, the proportions of patients experiencing diabetes-related complications, a composite of macrovascular complications, and a composite of microvascular complications were 40.5, 6.0, and 37.8%, respectively (Table 2). The group with treatment intensification within six months presented with significantly higher rates of stroke and DN when compared with the group with no delayed treatment intensification (HR 3.15; 95% CI 1.02–9.78 and HR 1.92; 95%CI 1.13–3.28, respectively). Treatment intensification after six months resulted in higher DN than no delayed treatment intensification, but this was not significant (HR 1.10; 95%CI 0.67–1.80). 

After controlling for confounding factors, the group with treatment intensification within six months had significantly higher rates of DN compared with the group with no delayed treatment intensification (adjusted HR 2.35; 95% CI 1.35–4.09). On the other hand, the group with treatment intensification after six months showed no statistically significant increase in DN (Table 3).

### 3.3. Effect of Treatment Intensification on HbA1c Levels

After treatment intensification, the mean ± SD values for HbA1c were 8.1 ± 1.6, 8.2 ± 1.3, and 8.0 ± 1.2 in the groups with no delayed treatment intensification, treatment intensification within six months, and treatment intensification after six months, respectively. The HbA1c values after treatment intensification among the three groups were not significantly different (*p*-value = 0.652). 

The group with no delayed treatment intensification presented a significant reduction in mean HbA1c from the baseline (*p*-value < 0.001), but the reductions in the groups with treatment intensification within six months and treatment intensification after six months were not significant (Table 4).

## 4. Discussion

This study comprised a post hoc analysis of the data from our previous study in order to analyze the effect of time to treatment intensification on diabetes-related complications. Members of the group that received treatment intensification within six months with HbA1c levels over the target had significantly increased DN rates when compared with the group with no delayed treatment intensification. On the contrary, the group with treatment intensification after six months revealed a trend of higher DN rates, but the difference was not statistically significant. Undetermined factors other than the time to treatment intensification may be responsible for complications. 

Regarding the risk of microvascular complications, the results of our study are consistent with a large representative cohort reporting that over four years, more stringent glucose-control measures were related to a lower risk of albuminuria onset or progression, but not with a meaningful reduction in the risk of MI [24]. In addition, the findings are consistent with the results of ADVANCE, a large randomized controlled trial. The ADVANCE study revealed that the intensive group significantly reduced DN (*p* = 0.006) after a median of five years’ follow-up but did so without affecting retinopathy (*p* = 0.50) compared with the standard group [25]. However, in the present study, treatment intensification after six months did not affect DN rates.

The findings in the present study contrast with those of a related study regarding the risk of macrovascular complications. An earlier cohort study reported that patients with newly diagnosed T2DM with a delay in treatment intensification by one year in conjunction with poor glycemic control presented significantly increased risk of MI, HF, stroke, and composite cardiovascular events (CVE) [18]. This outcome may be the result of uncontrolled blood sugar. Intensive glycemic control is claimed to be a factor associated with a lower risk of macrovascular complications [26,27]. 

Our study found that the group with no delayed treatment intensification tended to have higher baseline HbA1c levels than the delayed treatment intensification groups. The group with no delayed treatment intensification had a significantly higher baseline HbA1c than the group with treatment intensification after six months but did not have a significantly higher level than the group with treatment intensification within six months. HbA1c levels after treatment intensification among the groups with no delayed treatment intensification, treatment intensification within six months, and treatment intensification after six months were insignificant. Moreover, the difference in HbA1c after treatment intensification compared with the baseline HbA1c in the group with no delayed treatment intensification was significant (*p*-value < 0.001), but this difference was not found in the group with treatment intensification within six months or the group with treatment intensification after six months.

Thus, receiving treatment intensification within six months may lead to diabetes-related complications more often than when there is no delay in treatment intensification because patients in the former group have prolonged high blood sugar. Prolonged high blood sugar can lead to diabetes-related complications, especially microvascular complications [25,28,29,30]. Surprisingly, our study found an association with DN only in the group with treatment intensification within six months. This may have been due to DN events occurring adequately to produce statistical significance. 

The ADA 2022 guidelines recommend that patients with T2DM who do not meet the target levels should not delay treatment intensification, but data on the time to treatment intensification are unclear. To the best of our knowledge, this is the first study to investigate the effect of time to treatment intensification on diabetes-related complications in patients who are younger and have few comorbidities. Our findings are that delaying treatment intensification by even six months may lead to diabetes-related complications, especially DN.

According to the study’s findings, we suggest that healthcare providers intensify treatment when blood glucose levels do not achieve target levels. Related studies have shown that treatment intensification could decrease blood sugar levels [11,17] and that a 1% reduction in HbA1c significantly decreases the likelihood of DN [17]. The findings of a related study are consistent with those of our study in that treatment intensification within six months was associated with DN. Thus, patients with T2DM who have high blood glucose levels should receive treatment intensification for the target achievement of intermediate outcomes to reduce diabetes-related complications. Poor glycemic control is one factor that increases diabetes-related complications [31].

Our study encountered six limitations that should be acknowledged. Firstly, this study was retrospective and was conducted in real healthcare situations. The results should be interpreted cautiously due to residual confounders and missing data. Secondly, this study was a post hoc analysis of our published study. Therefore, the sample size was not calculated. The limited number of participants included in this study may have partly resulted in insufficient power to find statistical significance between the outcomes of each group. Moreover, the number of patients in each group was quite different. Thirdly, patients admitted to other hospitals with MI, HF, or stroke had unavailable data, which could have resulted in an underestimated complication rate. Fourthly, the effect of glucose control on diabetes-related complications could have been influenced by the length of follow-up; a longer follow-up period could alter these outcomes. Fifthly, we were unable to gather information on dietary habits, which are one of the risk factors for diabetes-related complications. Finally, the results from this study may show limited generalizability compared to those of the general population because of the differences among the included patients. Our study included patients with T2DM aged 40–65 years, patients with fewer comorbidities, and patients whose T2DM was less severe. In addition, patient characteristics, the availability of treatment options, definitions of treatment intensification, and clinical practices in each country also limit the generalizability of these results. Based on this, future studies in other regions or populations should be investigated.

## 5. Conclusions

In conclusion, this study looked at real clinical practice in Thailand. Patients diagnosed with T2DM and aged 40 to 65 years with HbA1c ≥ 7.0% were included, and compared to the group with no delayed treatment intensification, delayed treatment intensification by even six months was associated with an increase in diabetes-related complications, especially diabetic nephropathy. Hence, we encourage healthcare providers to intensify treatment immediately to reduce diabetic nephropathy. 

## Figures and Tables

**Table 1:** Baseline Characteristics.

Characteristic	All (*n* = 686)	No Delayed Treatment Intensification (*n* = 521)	Intensification within 6 Months (*n* = 53)	Intensification after 6 Months (*n* = 112)	*p*-Value
Sex					
Female (%)	297 (43.3)	214 (41.1)	26 (49.1)	57 (50.9)	0.107
Male (%)	389 (56.7)	307 (58.9)	27 (50.9)	55 (49.1)	
Age (Mean ± SD)	53.6 ± 6.0	53.6 ± 6.1	52.1 ± 6.2	54.3 ± 5.8	0.092
Duration of T2DM (Median (IQR))	5 (3–6)	4 (3–6)	4 (2.5–6)	5 (3–6)	0.386
Current drinker (%)	71 (10.4)	53 (10.2)	2 (3.8)	16 (14.3)	0.120
Current smoker (%)	31 (4.5)	25 (4.8)	1 (1.9)	5 (4.5)	0.763
Hypertension (%)	487 (71.0)	372 (71.4)	42 (79.2)	73 (65.2)	0.166
Dyslipidemia (%)	415 (60.5)	309 (59.3)	36 (67.9)	70 (62.5)	0.441
Charlson comorbidity index					
1 (%)	539 (78.6)	403 (77.4)	47 (88.7)	89 (79.5)	0.154
2 (%)	147 (21.4)	118 (22.6)	6 (11.3)	23 (20.5)	
Diabetic nephropathy (%)	69 (10.1)	56 (10.8)	4 (7.6)	9 (8.0)	0.659
Diabetic retinopathy (%)	53 (7.7)	49 (9.4) *	2 (3.8)	2 (1.8) *	0.007
Cardiovascular disease (%)	53 (7.7)	35 (6.7)	3 (5.7)	15 (13.4)	0.065
Baseline HbA1c (Median(IQR))	7.9 (7.3–9.0)	8.0 (7.4–9.1) *	7.7 (7.3–8.5)	7.6 (7.3–8.6) *	0.009
Lipid profile					
Total cholesterol (Mean ± SD)	178.4 ± 50.0	179.2 ± 52.2	170.5 ± 36.4	177.6 ± 44.3	0.730
Triglyceride (Median(IQR))	118 (81–171)	116 (79–171)	121.5 (81–172)	118 (84–164)	0.907
HDL-c (Median(IQR))	45 (39–54)	46 (40–55)	43.5 (45–49)	44 (39–49)	0.066
LDL-c (Mean ± SD)	104.2 ± 36.1	103.5 ± 36.5	101.5 ± 35.5	108.7 ± 34.9	0.572
Blood pressure					
Systolic (Median (IQR))	134 (124–146)	133 (123–146)	135 (130–144)	135 (124–145)	0.640
Diastolic (Mean ± SD)	78.3 ± 10.0	78.1 ± 10.1	80.1 ± 9.5	78.4 ± 9.7	0.393
eGFR (Mean ± SD)	87.7 ± 32.4	86.3 ± 32.9	99.5 ± 27.6	86.3 ± 32.9	0.085
BMI (Mean ± SD)	26.9 ± 4.7	26.7 ± 4.6	26.8 ± 4.2	27.9 ± 5.4	0.085
The use of insulin (%)	97 (14.1)	81 (15.6)	4 (7.6)	12 (10.7)	0.166

* *p*-value < 0.05.

**Table 2:** Number of patients having diabetes-related complications.

	All (*n* = 686)	No Delayed Treatment Intensification (*n* = 521)	Intensification within 6 Months (*n* = 53)	Intensification after 6 Months (*n* = 112)
Diabetes-related complications	278 (40.5)	211 (40.5)	25 (47.2)	42 (37.5)
A composite of macrovascular complications	41 (6.0)	30 (5.8)	5 (9.4)	6 (5.4)
Myocardial infarction	11 (1.6)	10 (1.9)	0 (0.0)	1 (0.9)
Stroke	18 (2.6)	12 (2.3)	4 (7.6)	2 (1.8)
Heart failure	20 (2.9)	16 (3.1)	1 (1.9)	3 (2.7)
A composite of microvascular complications	259 (37.8)	195 (37.4)	24 (45.3)	40 (35.7)
Diabetic nephropathy	121 (17.6)	86 (16.5)	16 (30.2)	19 (17.0)
Diabetic retinopathy	182 (26.5)	142 (27.3)	13 (24.5)	27 (24.1)

**Table 3:** Association between time to treatment intensification and diabetes-related complications.

	Intensification within 6 Months		Intensification after 6 Months	
	HR (95%CI)	Adjusted HR (95%CI)	HR (95%CI)	Adjusted HR (95%CI)
Diabetes-related complications	1.22 (0.81–1.85)	1.39 (0.90–2.15)	0.91 (0.66–1.27)	0.97 (0.70–1.36)
A composite of macrovascular complications	1.62 (0.63–4.17)	1.93 (0.66–5.66)	0.96 (0.40–2.31)	1.01 (0.41–2.46)
Myocardial infarction	–	–	0.50 (0.06–3.92)	0.66 (0.08–5.38)
Stroke	3.15 (1.02–9.78)	2.32 (0.62–8.64)	0.79 (0.18–3.52)	0.72 (0.16–3.28)
Heart failure	0.60 (0.08–4.50)	0.99 (0.12–7.85)	0.89 (0.26–3.05)	1.14 (0.32–4.06)
A composite of microvascular complications	1.27 (0.83–1.95)	1.43 (0.92–2.24)	0.94 (0.67–1.33)	1.04 (0.74–1.48)
Diabetic nephropathy	1.92 (1.13–3.28)	2.35 (1.35–4.09)	1.10 (0.67–1.80)	1.17 (0.70–1.94)
Diabetic retinopathy	0.88 (0.50–1.55)	0.98 (0.53–1.79)	0.86 (0.57–1.29)	0.94 (0.62–1.42)

Reference group: no delayed treatment intensification.

**Table 4:** The difference in mean HbA1c between baseline HbA1c and HbA1c after treatment intensification.

	No Delayed Treatment Intensification (*n* = 521)	Treatment Intensification within 6 Months (*n* = 53)	Treatment Intensification after 6 Months (*n* = 112)	*p*-Value ^a^
Baseline HbA1c (median (IQR))	8.0 (7.4–9.1) *	7.7 (7.3–8.5)	7.6 (7.3–8.6) *	0.009
Baseline HbA1c (mean ± SD)	8.4 ± 1.3 *	8.0 ± 1.1	8.0 ± 1.0 *	0.005
HbA1c after treatment intensification (mean ± SD)	8.1 ± 1.6	8.2 ± 1.3	8.0 ± 1.2	0.652
*p*-value ^b^	<0.001	0.533	0.850	

^a^*p*-value was obtained via Analysis of Variance (ANOVA) or Kruskal–Wallis test, as appropriate; ^b^*p*-value was obtained using paired *t*-test to compare between mean baseline HbA1c and HbA1c after treatment intensification in the same group; * *p*-value < 0.05.

## Data Availability

The corresponding author can provide the data used in this study on reasonable request.
